# Marchiafava-Bignami Disease: A Case Report of a Reversible Cause of Dementia

**DOI:** 10.7759/cureus.73146

**Published:** 2024-11-06

**Authors:** Paulo Conceição, Tânia Lopes, Vasco Abreu, Ana Reinas

**Affiliations:** 1 Internal Medicine, Centro Hospitalar Universitário de Santo António, Porto, PRT; 2 Neuroradiology, Centro Hospitalar Universitário de Santo António, Porto, PRT

**Keywords:** chronic alcoholism, corpus callosum demyelination, demyelinating neurological disorder, marchiafava-bignami disease, neuroimaging, thiamine supplementation

## Abstract

Marchiafava-Bignami disease (MBD) is a rare neurological disorder predominantly associated with chronic alcohol use, characterized by demyelination and necrosis of the corpus callosum. The condition often presents with cognitive dysfunction, motor deficits, and altered consciousness, which can range from mild confusion to severe stupor. Early recognition and treatment are crucial for improving outcomes. We report a case of MDB that was diagnosed in a 43-year-old woman with a history of chronic alcohol use and a 15-day history of progressive neurological symptoms, including impaired speech, confusion, and inability to walk independently. Brain MRI findings were consistent with MBD, showing acute demyelination of the anterior corpus callosum. The patient was treated with high-dose intravenous thiamine and B vitamins, resulting in a gradual and significant improvement. After one month, she regained coherent speech and the ability to walk independently.

## Introduction

Marchiafava-Bignami disease (MBD) is a rare neurological disorder characterized by the demyelination and necrosis of the corpus callosum and adjacent subcortical white matter [1]. Named after two Italian physicians who first described it in 1903, MBD is primarily associated with chronic alcoholism [2].

The presentation of the disease can be acute, subacute, or chronic and typically manifests with interhemispheric syndrome symptoms, including confusion, disorientation, dysarthria, disturbances in movement coordination, and seizures, and also chronic cognitive impairment [3]. Neuroimaging, such as MRI, often reveals characteristic abnormalities in the corpus callosum.

The exact cause of MBD remains unclear, but it is believed to involve a combination of genetic predisposition, nutritional deficiencies, and alcohol-toxic effects on the brain [4]. There are few treatment options besides supportive care and abstinence from alcohol. Prognosis varies, with some patients recovering, while others may face progressive and irreversible neurologic decline [5].

## Case presentation

This is the case of a 43-year-old woman without any other medical history besides chronic alcohol use disorder (160 grams of alcohol per day). She presented with a 15-day history of imperceptible speech, confusion, and loss of ability to walk autonomously. She stopped drinking alcohol five days before the first medical observation. On examination, she was disoriented to time and space and had dysarthria with slurred and incoherent speech. She could not maintain attention or execute the digit span backward. She had reduced psychomotor movements without asymmetry, with a mild reduction in proximal and distal strength in both arms and legs, rated 4/5 bilaterally. There were no cranial nerve palsies. The deep tendon reflexes were considered normal, with 2+ grades in both upper and lower limbs according to the National Institute of Neurological Disorders and Stroke (NINDS) scale. The sensory examination showed intact light touch, pinprick, and temperature sensations. Proprioception and vibration were mildly diminished, especially in the lower extremities. The cerebellar examination showed mild dysmetria at finger-to-nose and heel-to-shin and severe dysdiadochokinesia. The gait was ataxic.

Blood work showed macrocytic anemia (hemoglobin 10.7 g/dL and mean globular volume 110.1 fL), folic acid deficit (<0,6 ng/mL), mild elevation of gamma glutamyl-transferase (131 U/L, where the normal range is between 6 and 39 U/L), and a mild albumin deficit (3.35 g/dL, where the normal range is between 3.5 and 5 g/dL). The rest of her baseline investigation was normal, including ions (sodium, potassium, phosphate, and magnesium), thyroid function tests, venereal disease research, and autoimmune antibodies.

The computed tomography (CT) scan of the brain noted enlargement of cerebrospinal fluid spaces, greater than expected for the patient’s age, secondary to decreased brain volume. No other significant changes in brain density were observed. The cerebrospinal fluid examination was unremarkable.

**Figure 1:**
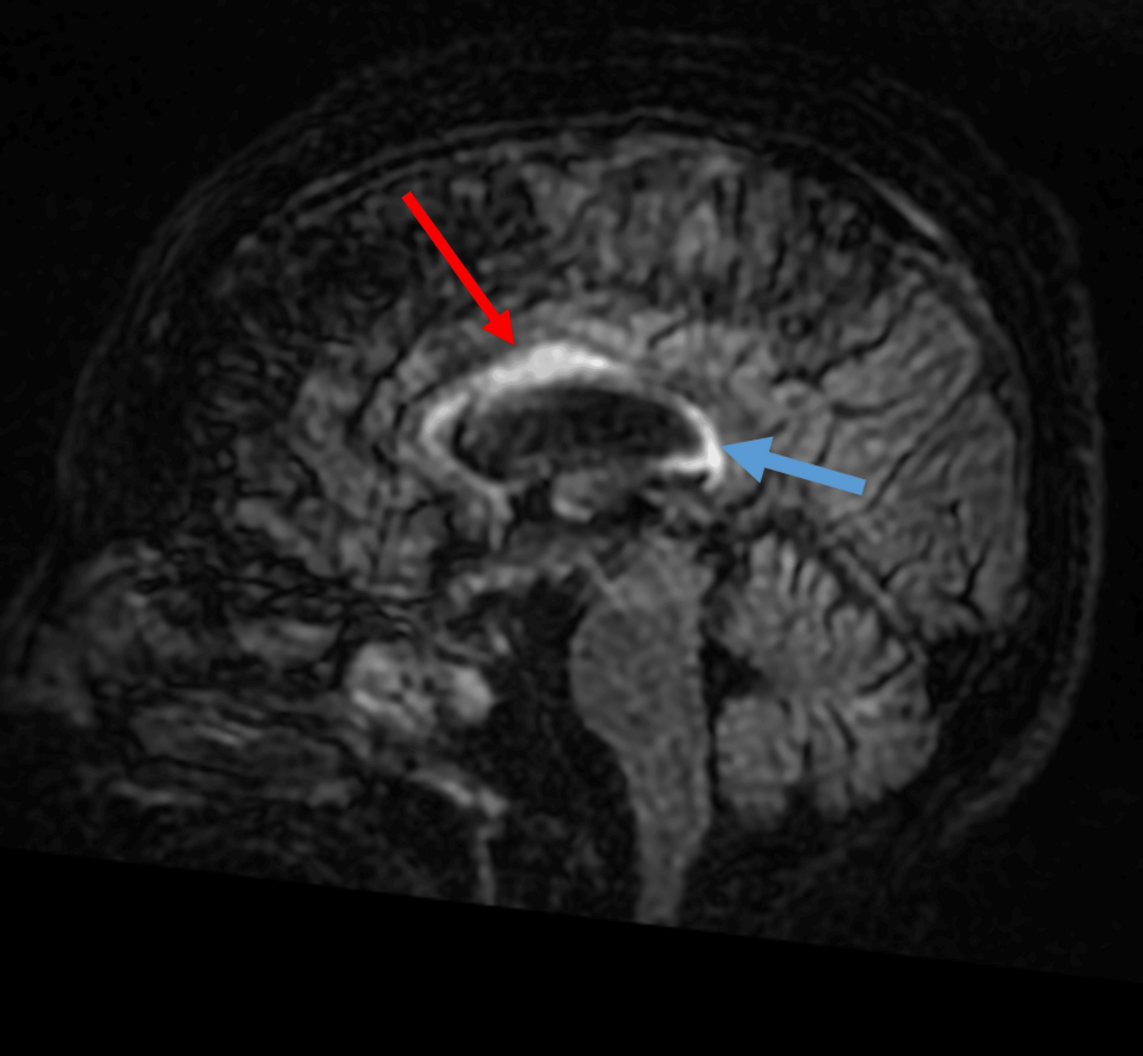
Acute demyelination due to Marchiafava-Bignami disease: MRI T2/FLAIR, sagittal MRI: Magnetic resonance imaging; FLAIR: fluid-attenuated inversion recovery Increased signal intensity affecting the anterior 2/3 of the corpus callosum, with slight associated expansion (red arrow), weaker but increased signal intensity in the splenium of the corpus callosum, but without expansive effect (blue arrow)

**Figure 2:**
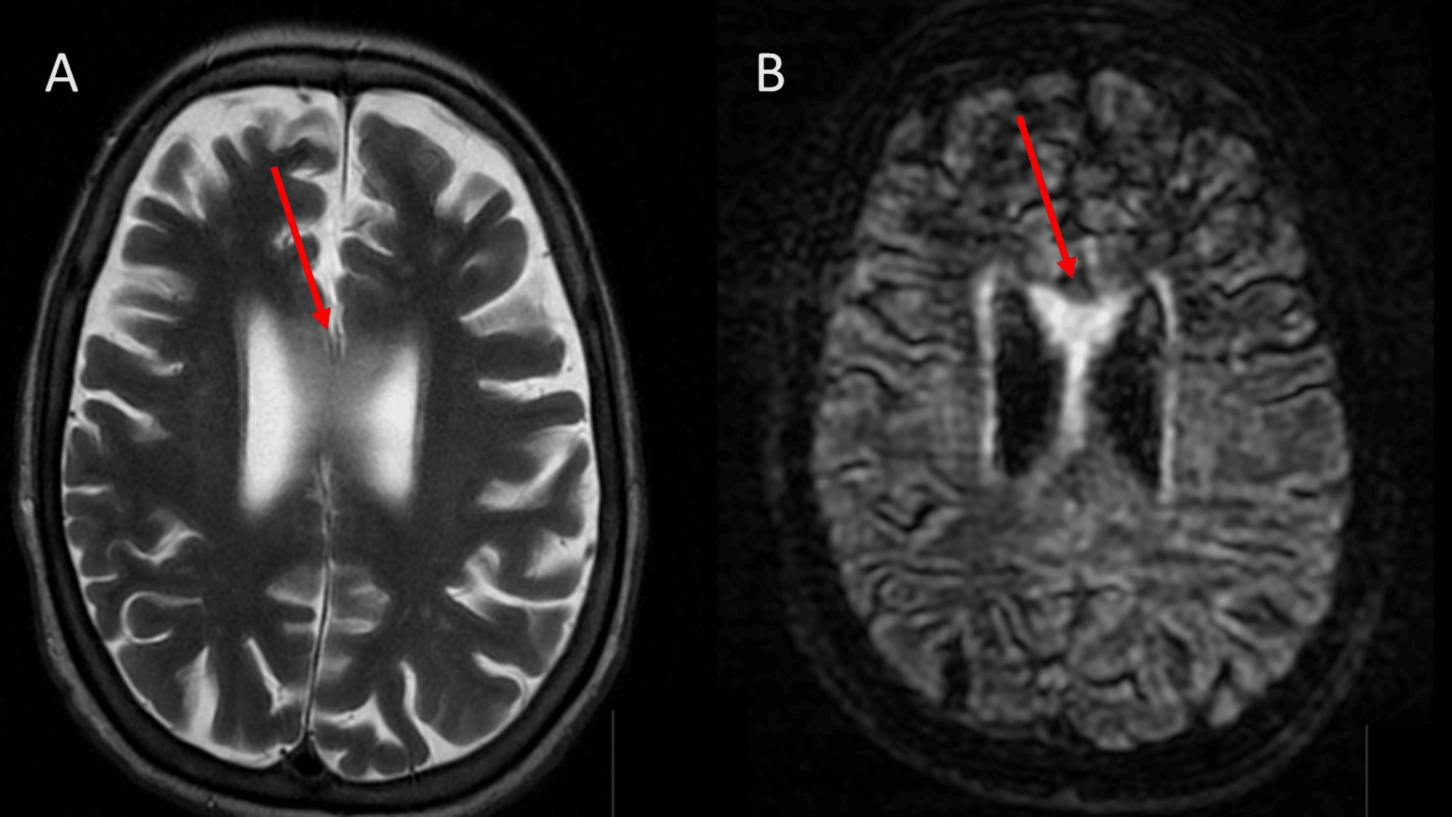
Acute demyelination due to Marchiafava-Bignami disease: T2 (A) and T2/FLAIR (B), axial MRI: Magnetic resonance imaging; FLAIR: fluid-attenuated inversion recovery Increased signal intensity affecting the anterior 2/3 of the corpus callosum, with slight associated expansion (red arrows)

**Figure 3:**
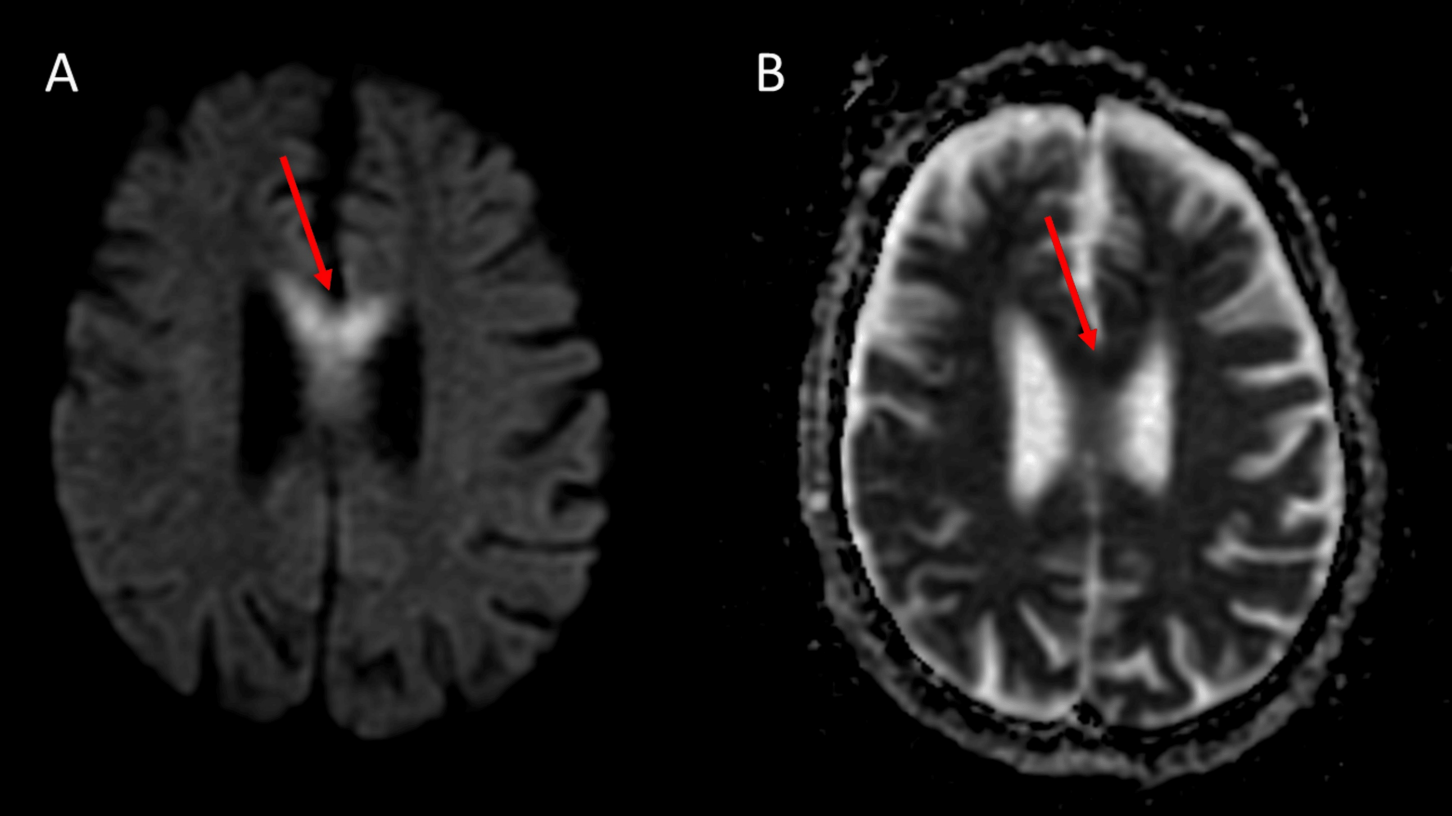
Acute demyelination due to Marchiafava-Bignami disease: diffusion-weighted image (A) and apparent diffusion coefficient map (B), axial Areas of diffusion restriction in the anterior 2/3 of the body of the corpus callosum (red arrows)

Brain MRI revealed high signal intensity lesions involving the anterior two-thirds of the corpus callosum on T2-weighted images (Figures 1-2) with mild expansion and more slight involvement of the splenium of the corpus callosum. On diffusion-weighted imaging (DWI) (Figure 3), there was restricted diffusion of the affected areas of the corpus callosum, revealing acute edema and demyelination. The neurological signs along with the MRI findings were suggestive of corpus callosum involvement due to MBD.

The patient’s body mass index at hospital admission was 13.74 kg/m^2^, and her nutrition risk screening 2002 was 3 points (positive for risk of malnutrition in hospitalized patients).

The patient started treatment with intravenous thiamine in a high dosage, starting with 500 mg three times daily for three days. After the first three days, it was continued with 250 mg of thiamine intravenously daily for five additional days. Following the intravenous treatment, thiamine was changed for oral formulation at the dose of 100 mg daily. Oral formulations of other B complex vitamins such as vitamins B6, B9 (folic acid), and B12 were administrated. An oral nutritional regime and a physical rehabilitation plan were applied.

Gradually, her mental state, speech, and gait started to improve under the vitamin supplementation and rehabilitation program. After one month of hospitalization, the patient was discharged to a rehabilitation facility to continue her treatment and rehabilitation. At this time, the patient showed marked improvement in speech, although mild dysarthria persisted. She was able to feed herself independently but still required assistance with hygiene care. She was able to bear weight but still needed support for walking.

Two months after discharge, the patient presented in consultation without dysarthria, revealing a coherent and fluid speech, and full recovery of the ability to walk again. Until that point, the patient had been on a daily dose of 100 mg of oral thiamine.

## Discussion

MBD is a rare disease highly associated with chronic alcoholism, although there are few cases reported in malnourished nonalcoholic individuals [6]. MBD clinical stages might be categorized as acute, subacute, or chronic, with an array of signs and symptoms present in each stage. Sudden neurological symptoms, including confusion, aggression, and seizures characterize the acute stage. Subacute (where our patient fits in) features persistent cognitive and motor deficits such as dysarthria, apraxia, and ataxia. The chronic stage involves long-term neurological impairments with severe global dementia, behavioral abnormalities, and visual hallucinations [1,7].

In the past, the diagnosis was only made postmortem. With the MRI breakthrough, neuroimaging is the current gold standard for diagnosis, revealing the typical corpus callosum demyelination during acute stages, or even necrosis and atrophy in chronic cases. A CT scan can sometimes detect changes related to MBD, showing areas of low density in the corpus callosum that suggest potential damage. However, it is generally less sensitive than MRI, and these changes are often subtle, making them easy to miss. A definitive diagnosis of MBD relies primarily on MRI. In addition to imaging, the patient's clinical history and symptoms support the diagnosis.

Wernicke-Korsakoff syndrome (WKS) is an important differential diagnosis when evaluating MBD, as both conditions are linked to chronic alcoholism and nutritional deficiencies. They share clinical signs, including cognitive impairment, confusion, and ataxia, but each has distinct features. WKS commonly presents with a classic triad of confusion, ataxia, and ophthalmoplegia, while MBD may include dysarthria, limb hypertonicity, and, in severe cases, coma. Imaging findings further help differentiate the two diseases. MBD primarily affects the corpus callosum, showing characteristic lesions in this area on MRI, whereas WKS typically involves the thalamus, hypothalamus, the periaqueductal region, and mammillary bodies [8]. The distinction in affected brain areas, visible on MRI, combined with specific clinical signs, is key to accurately distinguishing one condition from the other.

To date, there are no therapy guidelines for this disease. However, treatment with high doses of thiamine and other B complex vitamins gathers the most consensus [9]. Despite the lack of evidence, some cases use vitamin C and E as treatment due to their neuroprotective and antioxidant properties [10]. Other case reports used high doses of corticosteroids, even though the evidence is also limited [11].

The prognosis may differ between patients with MBD. While some may acquire full recovery, others may sustain symptoms. In extreme situations, this disease may lead to death, due comatose state [12]. One believes this is a successful case once there was no delay in the diagnosis and proper administration of high doses of thiamine allowing, in a few months, the patient full recovery.

## Conclusions

MBD is a rare but crucial differential diagnosis in chronic alcoholics presenting with rapidly progressive cognitive decline. This disorder, often under-recognized, requires clinical vigilance to identify its wide spectrum of signs, symptoms, and distinctive MRI findings, which are key to early diagnosis. Despite the lack of standardized treatment guidelines, aggressive vitamin supplementation, particularly with thiamine, remains the primary therapeutic approach due to the likely involvement of nutritional deficiencies. Prognosis varies significantly: while some patients respond to treatment with noticeable cognitive and motor improvements, others may experience irreversible neurological impairment. This variability underscores the need for further research to refine diagnostic criteria and develop evidence-based treatment protocols that may improve patient outcomes. Greater clinical awareness and study of MBD will be essential to reducing the rate of misdiagnosis and improving care for those affected by this serious condition.

## References

[1] Tian TY, Pescador Ruschel MA, Park S, Liang JW (2024). Marchiafava-Bignami Disease. StatPearls. Treasure Island.

[2] Wenz H, Eisele P, Artemis D, Förster A, Brockmann MA (2014). Acute Marchiafava-Bignami disease with extensive diffusion restriction and early recovery: case report and review of the literature. J Neuroimaging.

[3] Hillbom M, Saloheimo P, Fujioka S, Wszolek ZK, Juvela S, Leone MA (2014). Diagnosis and management of Marchiafava-Bignami disease: a review of CT/MRI confirmed cases. J Neurol Neurosurg Psychiatry.

[4] Fernandes LM, Bezerra FR, Monteiro MC (2017). Thiamine deficiency, oxidative metabolic pathways and ethanol-induced neurotoxicity: how poor nutrition contributes to the alcoholic syndrome, as Marchiafava-Bignami disease. Eur J Clin Nutr.

[5] Matsuura H, Shindo K (2018). Marchiafava-Bignami disease. QJM.

[6] Caulo M, Briganti C, Notturno F (2009). Non-alcoholic partially reversible Marchiafava-Bignami disease: review and relation with reversible splenial lesions: a case report and literature review. Neuroradiol J.

[7] Singh S, Wagh V (2022). Marchiafava Bignami disease: a rare neurological complication of long-term alcohol abuse. Cureus.

[8] Wijnia JW (2022). A clinician's view of Wernicke-Korsakoff syndrome. J Clin Med.

[9] Latt N, Dore G (2014). Thiamine in the treatment of Wernicke encephalopathy in patients with alcohol use disorders. Intern Med J.

[10] Zhang Y, Culpepper K, Mathew R, CruzSaavedra L (2022). Marchiafava-Bignami disease presenting as reversible coma. BMJ Case Rep.

[11] Singer E, Bhatt K, Prashad A, Rudman L, Gadelmoula I, Michel G (2023). Diagnosis and management of Marchiafava-Bignami Disease, a rare neurological complication of long-term alcohol abuse. Discoveries (Craiova).

[12] Dong X, Bai C, Nao J (2018). Clinical and radiological features of Marchiafava-Bignami disease. Medicine (Baltimore).

